# Ammonium Fluoride Passivation of CdZnTeSe Sensors for Applications in Nuclear Detection and Medical Imaging

**DOI:** 10.3390/s19153271

**Published:** 2019-07-25

**Authors:** Stephen U. Egarievwe, Utpal N. Roy, Carmella A. Goree, Benicia A. Harrison, Jeanette Jones, Ralph B. James

**Affiliations:** 1 Nuclear Engineering and Radiological Science Center, Department of Electrical Engineering and Computer Science, Alabama A&M University, Normal, AL 35762, USA; 2 National Security and Nonproliferation Department, Brookhaven National Laboratory, Upton, NY 11973, USA; 3 Biological and Environmental Sciences Department, Alabama A&M University, Normal, AL 35762, USA; 4 Center for Biomedical, Behavioral and Environmental Health Research, Alabama A&M University, Normal, AL 35762, USA; 5 Science and Technology Directorate, Savannah River National Laboratory, Aiken, SC 29808, USA

**Keywords:** CdZnTeSe detectors, chemical passivation, gamma-ray detector, nuclear radiation detector, X-ray detector

## Abstract

Cadmium zinc telluride selenide (Cd_1−x_Zn_x_Te_1−y_Se_y_ or CZTS) is one of the emerging CdTe-based semiconductor materials for detecting X- and gamma-ray radiation at or near room temperature (i.e., without cryogenic cooling). Potential applications of CZTS sensors include medical imaging, X-ray detection, and gamma-ray spectroscopy. Chemical passivation of CZTS is needed to reduce the conductivity of Te-rich surfaces, which reduces the noise and improves the device performance. In this study, we focus on the effect of surface passivation of CZTS using a 10% aqueous solution of ammonium fluoride. The effects of the chemical treatment were studied on the leakage current, charge transport measured as the electron mobility-lifetime (µτ) product, and the spectral resolution measured as the full-width at half-maximum (FWHM) of specific peaks. After passivation, the leakage current increased and began to decrease towards pre-passivation levels. The energy resolutions were recorded for eight applied voltages between −35 V and −200 V. The results showed an average of 25% improvement in the detector’s energy resolution for the 59.6 keV gamma peak of Am-241. The electron µτ product was unchanged at 2 × 10^−3^ cm^2^/V. These results show that ammonium fluoride is effective for chemical passivation of CZTS detectors.

## 1. Introduction

Cadmium zinc telluride selenide (CdZnTeSe or CZTS) has shown great potential as a quaternary semiconductor compound of cadmium telluride (CdTe) based materials for detecting nuclear radiation at room temperature (i.e., without cryogenic cooling) [1,2,3]. The ability of CdTe-based semiconductor nuclear detectors to operate at room temperature has advantages that include (1) reduction in the production and operational costs of the detection system, (2) fabrication into portable devices, and (3) remote battery-operated deployment applications. Cadmium zinc telluride (CdZnTe or CZT) is the most prominent CdTe-based room temperature nuclear radiation detector material with applications in medical imaging, nuclear nonproliferation, astrophysics, and spectroscopy [4,5,6,7]. Even with the advances in the development of CdZnTe over the past two to three decades, the material is still prone to defects that are related to large numbers of Te inclusions, subgrain boundaries, and compositional nonuniformity [8,9,10,11,12]. These defects trap charges generated by absorption of radiation, thus limiting the charge transport abilities of the detector and resulting in lowering its performance for precise measurements of X- and gamma-ray energies [8]. This is especially critical in applications that require large-volume detectors [8,13]. These defects (Te inclusions and subgrain boundary networks) are nonuniformly distributed in the CZT matrix. This results in spatial variation of charge transport properties in CZT and is responsible for the spreading of pulse height in large-volume detectors [13]. This reduces detector resolution. The major advantages of CZTS include increased compositional uniformity, less Te inclusions, and absence of subgrain boundary network [1,3]. Thus, the CZTS potentially offers uniformity in spatial charge transport properties. The spatial charge transport uniformity will result in increasing the performance and yield of high-quality detectors, hence lowering the cost of high-resolution devices. In addition, compositional homogeneity can increase the overall yield of detector-grade material in CZTS as compared with CZT [1,14,15].

The major stages of processing a wafer for device fabrication of CdTe-based detectors include cutting, polishing, chemical etching, contact deposition, and chemical passivation [16,17,18,19,20]. The chemical etching and passivation of CZTS sensors for medical imaging, X-ray detection, and gamma-ray spectroscopy applications are needed to reduce the conductivity of Te-rich wafer surfaces that remain after bromine-methanol etchants and to improve the efficiency. The chemical treatment of the CdTe-based detector wafers produces a smoother and more stoichiometric surface after polishing [20]. It also minimizes the oxidation of the wafer surfaces, thus leading to an increased shelf life of the detector [21,22]. 

The present study covers the effects of surface passivation of CZTS using an ammonium fluoride solution. The effects of the chemical treatment were studied on the leakage current, the charge transport measured as the electron mobility-lifetime (µτ) product, and the spectral resolution of the detector for incident gamma rays.

## 2. Materials and Methods

The experimental procedure involved a set of measurements that includes current-voltage (I-V), charge transport, and energy resolution, on a CZTS sensor (or detector) before and after passivation in ammonium fluoride (NH_4_F) solution. The same sample was used for both sets of measurements. The sample preparations are the same for both sets except for the passivation in NH_4_F solution, which is added to the fabrication of the sample for the second set of measurements.

### 2.1. Detector Material and Composition

**Figure 1:**
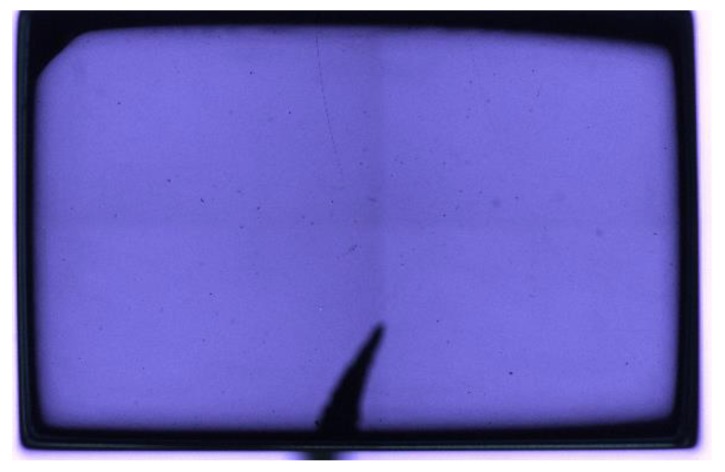
Infrared transmission image (7.00 mm × 4.65 mm) of the cadmium zinc telluride selenide (CZTS) sample, after polishing the surfaces to a mirror-shine finish. The image (composed from four images) was obtained with a Nikon Eclipse LV100 microscope fitted with an infrared light source and camera, a motorized stage with xyz-translation capability, and a software for capturing and analyzing images. The object protruding from the lower side is used to mark and track the wafer placement orientation.

The CdZnTeSe used in this study was grown by the Traveling Heater Method (THM) with a material composition of Cd_1−x_ Zn_x_Te_1−y_Se_y_ where x = 0.1 and y = 0.04. It was doped with indium. The growth process was similar to that reported by Roy et al. [15], which started with the synthesis of the CdZnTnSe from predetermined stoichiometric amounts of 6N-purity CdZnTe and CdSe. The inner walls of the conically-tipped quartz ampoules, which were used for the synthesis and THM growth, were carbon coated. The carbon coatings were made by cracking spectroscopic-grade acetone at a temperature of about 900 °C. The coated ampoules were annealed for 1 hour at a temperature of about 1150 °C. The CZTS was grown in a Te-rich solution. The tellurium and indium were of 6N purity. The THM process used in this study was carried out in a 3-zone furnace. Ingots were grown by lowering the ampoules a few millimeters per day [15]. The wafer used in this experiment was cut from the as-grown CZTS ingot using a diamond-impregnated wire saw. The wafer’s dimensions were 7.00 × 4.65 × 2.70 mm^3^. An infrared transmission image of the sample is shown in Figure 1. 

The infrared image in Figure 1 is a composite of four images automatically stitched together by the software that comes with the Nikon Eclipse LV100 microscope used for the imaging. The CZTS matrix is transparent to infrared light, while Te inclusions are opaque and appear as dark spots in transmission. Figure 1 shows that the CSTS wafer is almost free of Te inclusions. This is good for charge-collection uniformity and radiation detection efficiency, since Te inclusions trap charge carriers [8].

### 2.2. Planar Detector Fabrication and Passivation

The wafer cut from the CZTS ingot for fabrication into a planar detector was polished using silicon carbide abrasive papers. The wafer was first lapped with 800 grit silicon carbide paper. This was followed by subsequent polishing in 1000 grit and 1200 grit papers. The surfaces of the wafer were further smoothed by successively polishing on MultiTex pads with alumina powder of varying sizes (from 3.0 µm to 0.1 µm). The wafer was rinsed in distilled water to remove residues from the polishing process. Compressed air was used to dry the wafer. Gold electrical contacts where deposited on the two opposite sides of the wafer using an electroless deposition technique [16,23,24,25]. This process involved pipetting gold chloride (AuCl_3_) solution on opposite sides of the wafer. After reaction with the surface, excess AuCl_3_ solution was removed using a felt paper. The current-voltage (I-V), charge-transport, and detector energy resolution experiments where then performed on the sample.

To study the effects of surface passivation using NH_4_F, the gold contacts were removed by using MultiTex pads with a 3.0 µm alumina power. The surfaces were then smoothened by successive fine polishing with smaller sizes of alumina power until reaching a 0.1 µm powder size. The sample was then rinsed in distilled water and dried using compressed air. The passivation process was accomplished by dipping the wafer in a 10% by weight of aqueous solution of NH_4_F in three consecutive dips for five minutes. The sample was then dried, and gold contacts where deposited as previously described. The measurement experiments were then repeated on the passivated sample.

### 2.3. Current-Voltage, Charge-Transport, and Detector Energy Resolution Experiments

Current-voltage measurements were carried out in a specially customized aluminum box equipped with a Keithley Picoammeter, Voltage Source, model number 6487. The applied voltage range was −500 V to 500 V. The I-V data were collected for the sample before passivation in NH_4_F, and repeated 4, 10, 21, and 28 days after passivation. 

A special sample holder by eV Products (now Kromek) was used in the experiment to record the response of the detector to a sealed Am-241 nuclear radiation source. The sample holder was made of brass and had a beryllium window where the radiation source was placed. The signal generated by the radiation in the detector was passed through a preamplifier, an amplifier, and a multichannel analyzer (MCA) to a computer. The shaping time for all the detector measurements were kept at 3 µs. The charge-transport was determined as the electron mobility-lifetime (µτ) product via recording of the charge collection at various applied voltages and fitting to the Hecht equation. The energy resolution for the 59.6 keV gamma peak of Am-241 was measured as the full-width at half-maximum (FWHM) of the peak using commercial software provided with the MCA.

## 3. Results and Discussion

A previous study of the ternary compound CdZnTe showed that NH_4_F affects the I-V curve and the energy resolution of the detector [16]. Some similarities were observed for the material, CdZnTeSe, used in this study, which was developed by the addition of Se to CdZnTe. Chemical treatment and passivation are surface processes that affect the surface species of the material. The species that dominate the surfaces of CdTe-based materials are Cd, Te, and TeO_2_, and their quantities and ratios vary according to the surface stoichiometry and chemical solutions used in the passivation [16]. The CdZnTe study showed that the quantity of Te present on the surfaces is higher than that of TeO_2_, and this is reversed after passivation in NH_4_F solutions [16]. In other chemical treatment studies, the chemo-mechanical polishing of CdZnTe surfaces with a solution of bromine, methanol, ethylene, and glycol decreased the quantity of Te and increased that of TeO_2_ but did not reverse the ratios [17]. In addition, chemo-mechanical polishing in a mixture of hydrogen bromide in hydrogen peroxide and ethylene glycol solutions does not reverse the TeO_2_/Te ratio but increases TeO_2_ while decreasing Te [17].

### 3.1. Current-Voltage Characteristics

**Figure 2:**
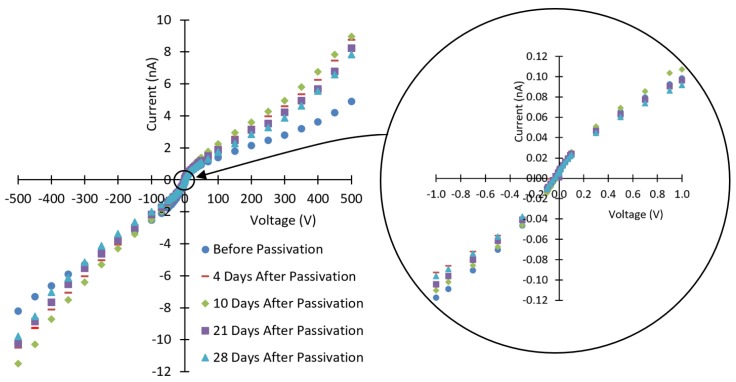
The current-voltage characteristics of the CdZnTeSe detector before and after passivation. The electrical resistivity was calculated to be ~1.3 × 10^10^ Ω-cm. The currents increased after passivation and then decreased as the passivated surfaces stabilize.

The I-V curves for the CZTS detector before and after passivation are shown in Figure 2. The resistivity of the wafer was calculated to be 1.3 × 10^10^ Ω-cm. The passivated sample was left to stabilize for four days before the I-V measurements. 

**Figure 3:**
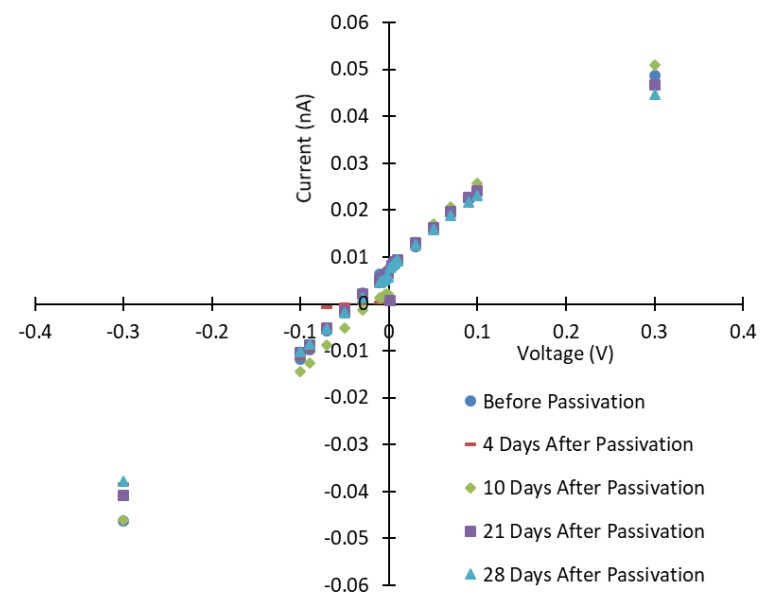
Current-voltage graph for very low voltages in the range −0.3 V and 0.3 V.

**Figure 4:**
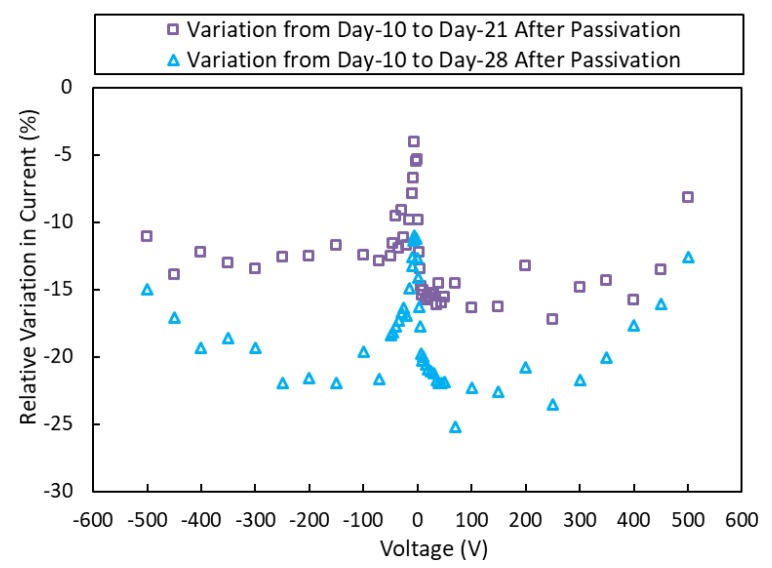
Relative variations in currents for days 10–21 and days 10–28 after passivation. These show a continuous decrease from day 10.

The leakage currents were increased after passivation and then decreased afterwards. Similar results were reported for CdZnTe [16]. The I-V curves after 10, 21, and 28 days show a continuous decrease in the currents as the passivated surfaces of the CZTS wafer continue to stabilize, except for positive and negative applied voltages below 0.1 V (see Figure 2 and Figure 3). Figure 4 shows the relative variations in currents for days 10–21 and days 10–28. These variations show continuous decrease from day 10 after passivation. The measured currents result from the bulk currents and the surface currents, and any changes in the measured currents come from changes in the surface currents [16].

### 3.2. Charge Transport

**Figure 5:**
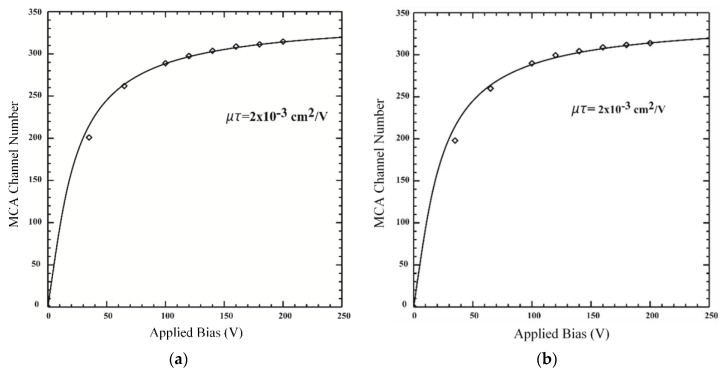
The plots of MCA channel number at various applied voltages: (**a**) Before passivation in NH_4_F solution, (**b**) after passivation in NH_4_F solution.

The electrons and holes generated in the CZTS wafer by incoming radiations are driven toward the respective anode or cathode by the internal electric field created by the applied voltage. The more the number of charge carriers collected at the electrode and the higher the charge-transport uniformity, the better the detector performance. The charge transport is measured in this experiment as an electron mobility-lifetime (µτ) product. Figure 5 shows the plot of the MCA channel number at various applied voltages for the sample before and after passivation. The electron µτ-product is 2 × 10^−3^ cm^2^V^−1^ for both cases. This implies that the passivation does not significantly affect the charge transport properties of the detector matrix as measured by the µτ-product. The µτ-product is important in the detector’s efficiency. There are other factors that also affect the energy resolution of the CdTe-based semiconductor detectors. The main factors that determine the energy resolution are electronic noise and fluctuations in the charge carriers (electron-hole pairs) produced by incoming ionizing radiation and collected at the electrodes [26]. The passivation process could affect these factors due to changes in the surface stoichiometry. According to Wright et al. [27], the resistivity of the region near the surface could change the electric field in the detector matrix. Changes in the internal electric fields of chemically treated CdZnTe samples were also reported by Pekarek et al. [28]. 

### 3.3. Energy Resolution

**Figure 6:**
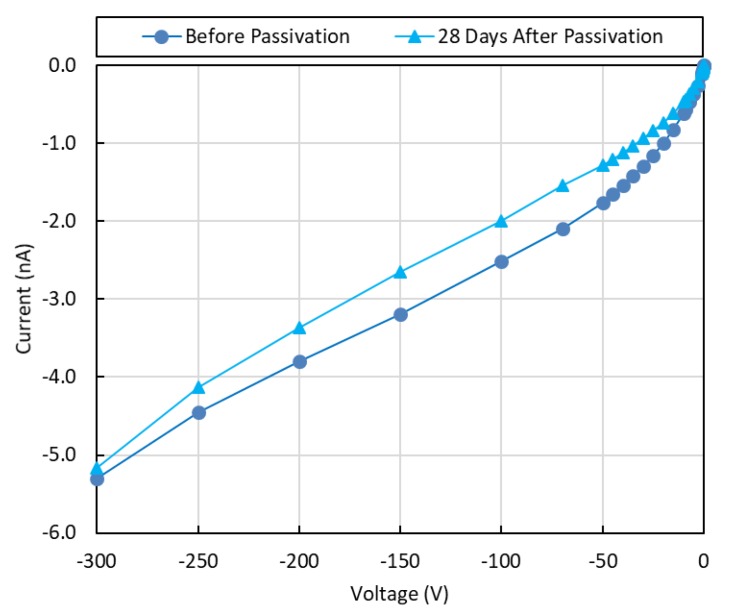
The applied voltages for the CZTS detector were selected from the negative bias range where the absolute values of the currents were lower than pre-passivation values.

The chemical passivation of CdTe-based detectors causes changes to the compositional stoichiometry at the surfaces, particularly those of Cd, Te, and TeO_2_ [16,17]. Stoichiometric changes at the surfaces, including the metal contact and detector interface, could lead to changes in the electron-hole recombination rates at the surface [27]. High leakage currents result in high electronic noise. Thus, our applied bias range was chosen from the voltage region where the currents were lower, 28 days after passivation as compared with pre-passivation values (see Figure 6).

**Figure 7:**
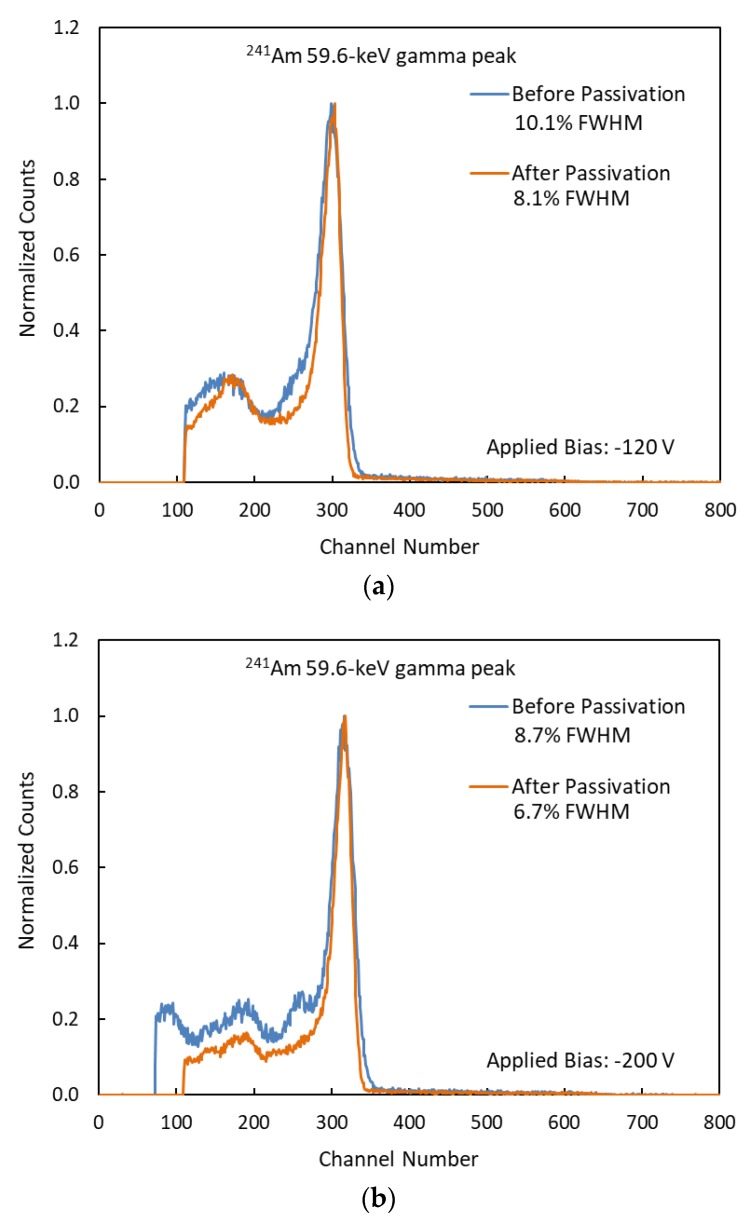
CZTS detector responses to Am-241 source before passivation and 28 days after passivation. The shaping time is 3 µs. (**a**) Detector responses at −120 V. (**b**) Detector responses at −200 V.

The negative sign on the negative currents represents the direction of electric current. Thus, we must use the absolute values of the currents when discussing their contributions to the electronic noise. Hence, a closer look at Figure 2 shows that 28 days after passivation in NH_4_F, the leakage currents (absolute values) for voltages between −300 V and 1 V were lower than pre-passivation values (see Figure 6 for applied voltages of 0 to −300 V). Thus, the applied electric field used for detector operation over this bias-voltage range is expected to contribute less electronic noise after passivation. This could explain the improved energy resolution for the detector at this applied negative bias range (see Figure 7 and Table 1).

The responses of the detector, before and after passivation, to a sealed Am-241 nuclear radiation source, shown in Figure 7, indicate improvement in energy resolution. The improvement on the energy resolutions for the 59.6 keV gamma peak at various applied voltages are shown in Table 1. For each applied bias, there is no significant change in the 59.6 keV gamma peak position before and after passivation. The experiments showed an improvement in the energy resolution for each applied bias. The applied bias of −200 V gave the best energy resolution (8.7% FWHM) prior to passivation in NH_4_F. After passivation, the best energy resolution was observed at −180 V (6.4% FWHM). The average improvement in energy resolution was 25%. The maximum improvement was 33% at −35 V, and the minimum improvement was 19% at −100 V.

## 4. Conclusions

We have studied the effects of surface passivation for CZTS using a 10% aqueous solution of ammonium fluoride. The effect of the chemical treatment on the leakage current is an increase after passivation and then a decrease as the surfaces stabilize. No change was observed for the charge transport properties, as measured by the electron µτ-product. Improvements in energy resolutions were recorded for the eight applied voltages of −35, −65, −100, −120, −140, −160, −180, and −200 V. These voltages are within the range where the absolute values of leakage currents were lower 28 days after passivation as compared with pre-passivation values. The results showed an average of 25% improvement in energy resolution for the 59.6 keV gamma peak of Am-241. The lower leakage currents (absolute values) and the improvements in energy resolution at the operational voltages recorded in this experiment show that ammonium fluoride solution is an effective chemical for passivating CdZnTeSe detectors.

## Figures and Tables

**Table 1:** Energy resolutions for the 59.6 keV gamma peak of Am-241 at various applied voltages.

Applied Voltage (V)	FWHM before Passivation (%)	FWHM after Passivation (%)	Improvement in Energy Resolution
−35	17.9	12.0	33%
−65	12.9	10.0	22%
−100	9.9	8.0	19%
−120	10.1	8.1	20%
−140	10.0	7.2	28%
−160	9.3	6.9	26%
−180	8.9	6.4	28%
−200	8.7	6.7	23%
